# Functional segregation within the pelvic nerve of male rats: a meso‐ and microscopic analysis

**DOI:** 10.1111/joa.13221

**Published:** 2020-06-29

**Authors:** Martin M. Bertrand, Nadja Korajkic, Peregrine B. Osborne, Janet R. Keast

**Affiliations:** ^1^ Department of Anatomy and Neuroscience University of Melbourne Melbourne Vic. Australia; ^2^ Department of Visceral Surgery CHU de Nîmes Nîmes France; ^3^ Montpellier Laboratory of Informatics, Robotics and Microelectronics (LIRMM) ICAR Team, French National Centre for Scientific Research (CNRS) Montpellier University Montpellier France

**Keywords:** autonomic ganglion, bioelectronic medicine, inferior hypogastric plexus, parasympathetic, pelvic ganglion, splanchnic nerve, sympathetic, urinary tract, visceral nerve

## Abstract

The pelvic splanchnic nerves are essential for pelvic organ function and have been proposed as targets for neuromodulation. We have focused on the rodent homologue of these nerves, the pelvic nerves. Our goal was to define within the pelvic nerve the projections of organ‐specific sensory axons labelled by microinjection of neural tracer (cholera toxin, subunit B) into the bladder, urethra or rectum. We also examined the location of peptidergic sensory axons within the pelvic nerves to determine whether they aggregated separately from sacral preganglionic and paravertebral sympathetic postganglionic axons travelling in the same nerve. To address these aims, microscopy was performed on the major pelvic ganglion (MPG) with attached pelvic nerves, microdissected from young adult male Sprague–Dawley rats (6–8 weeks old) and processed as whole mounts for fluorescence immunohistochemistry. The pelvic nerves were typically composed of five discrete fascicles. Each fascicle contained peptidergic sensory, cholinergic preganglionic and noradrenergic postganglionic axons. Sensory axons innervating the lower urinary tract (LUT) consistently projected in specific fascicles within the pelvic nerves, whereas sensory axons innervating the rectum projected in a complementary group of fascicles. These discrete aggregations of organ‐specific sensory projections could be followed along the full length of the pelvic nerves. From the junction of the pelvic nerve with the MPG, sensory axons immunoreactive for calcitonin gene‐related peptide (CGRP) showed several distinct patterns of projection: some projected directly to the cavernous nerve, others projected directly across the surface of the MPG to the accessory nerves and a third class entered the MPG, encircling specific cholinergic neurons projecting to the LUT. A subpopulation of preganglionic inputs to noradrenergic MPG neurons also showed CGRP immunoreactivity. Together, these studies reveal new molecular and structural features of the pelvic nerves and suggest functional targets of sensory nerves in the MPG. These anatomical data will facilitate the design of experimental bioengineering strategies to specifically modulate each axon class.

## Graphical abstract

The pelvic nerve is a mixed sensory‐motor nerve and a potential target of neuromodulation for pelvic organ dysfunction. We have visualised neural components within its individual fascicles and identified segregation of sensory pathways innervating the lower bowel and urinary tracts. We have also defined new features of sensory connectivity in the pelvic ganglia. These anatomical data will facilitate the design of experimental bioengineering strategies to specifically modulate each axon class.

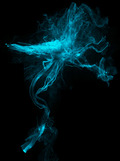

## 1 Introduction

In recent years, many new clinical applications have emerged for bioelectronics and neuromodulation (electrical modulation of neural properties) to regulate organ function (Birmingham *et al*. 2014; Horn *et al*. 2019; Moore *et al*. 2019; Payne *et al*. 2019). As the published reviews demonstrate, the vagus nerve has become a key neuromodulation target, with efficacy demonstrated on a range of thoracic and abdominal conditions, in addition to a growing number of CNS sites. The vagus is part of the ‘cranial’ component of the parasympathetic system and contains preganglionic axons that innervate numerous ganglia near and within many organs; however, as the majority of the axons within the vagus are sensory (Foley and DuBois, 1937; Prechtl and Powley, 1985; Prechtl and Powley, 1990; Berthoud and Neuhuber, 2000), the success of vagal neuromodulation over such diverse targets is ascribed to modulation of both its afferent and efferent components.

Whereas the vagus regulates abdominal and thoracic organs, the pelvic organs are instead primarily innervated by more caudal neural circuits that have also become a focus of neuromodulation (Gaunt and Prochazka, 2006; Liberman *et al*. 2017; Abello and Das, 2018; Kessler *et al*. 2019; Moore *et al*. 2019). Specifically, the ‘sacral’ component of the parasympathetic system, working together with sacral afferent pathways, is critical for micturition, defecation and sexual function; furthermore, sacral afferents also function in inflammatory and pain conditions originating in the pelvic organs (Gonzalez *et al*. 2014; de Groat and Yoshimura, 2015; Grundy *et al*. 2019). Several fundamental anatomical and functional principles of this sacral neuroregulatory system mirror the vagus, with the functionally homologous nerves being the pelvic splanchnic nerves (human) and pelvic nerve (rodent). These multi‐functional nerves contain axons of all parasympathetic preganglionic neurons that regulate pelvic organ function, synapsing on autonomic ganglion neurons that project to relevant tissues in the urinary or digestive tracts, or sex organs. In humans, these final motor neurons lie in a complex structure, the inferior hypogastric plexus, whereas in rodents they are aggregated in the major pelvic ganglia (MPGs). Similar to the situation in the vagus, there is also a large sensory component of the pelvic splanchnic and pelvic nerves, which carry the afferent axons from sacral dorsal root ganglia, that then innervate the pelvic organs.

Although not yet as extensive as the vagal neuromodulation field, sacral neuromodulation has already shown promising results for several clinical conditions, including fecal and urinary incontinence (Ripetti *et al*. 2002; Faucheron *et al*. 2012), constipation (Maeda *et al*. 2015), painful bladder syndrome/interstitial cystitis (Chai *et al*. 2000) and sexual dysfunction (Yih *et al*. 2013). Some aspects of the mechanism have been investigated (Amend *et al*. 2011; Gourcerol *et al*. 2011), but many gaps remain in our understanding. This has limited further improvements in efficacy or rational redesign for different clinical conditions and has driven increasing interest in functional mapping of the sacral neural pathways.

The most common clinical approach for sacral neuromodulation is to place an electrode in front of the left or right third sacral foramen (Matzel *et al*. 2017), providing the capacity to influence activity in two major neural projections, the pudendal (somatic) and pelvic splanchnic (visceral) nerves, that usually originate from the second to third sacral roots (Baader and Herrmann, 2003). This approach, therefore, has the potential to modulate a diverse range of tissues, including the striated muscle of sphincters and pelvic floor, and smooth muscle, epithelia and glandular tissues of the urogenital organs and lower bowel. Efficacy of this approach for modulation of visceral function via the pelvic splanchnic nerves has been demonstrated (Kenefick *et al*. 2003), however a strategy more specifically directed to the pelvic splanchnic nerves may be more successful for treating autonomic dysfunction or pain originating from the pelvic viscera. This more peripheral location for targeting neuromodulation shows considerable promise (de Groat and Tai, 2015; Brouillard *et al*. 2018; Peh *et al*. 2018) but remains under‐explored, even though the pelvic splanchnic nerves are accessible through laparoscopic dissection (Possover *et al*. 2007) and positioning of a neuromodulation device is feasible through a minimally invasive approach.

To drive mechanism‐based design of neuromodulation targeted to the pelvic viscera, it is important to define the properties of functionally distinct neural pathways that project within the pelvic splanchnic nerves. We aimed to address this issue in adult male rats, where the homologous nerve (pelvic nerve) has been characterised at the ultrastructural level (Hulsebosch and Coggeshall, 1982) and the target of sacral preganglionic axons, the MPG, has been characterised in most detail (Dail *et al*. 1975; Dail, 1996; Keast, 1999a; Keast, 2006). The pelvic nerve regulates all of the pelvic organs via its three major functional components: (1) sacral (parasympathetic) preganglionic axons that synapse on neurons in the MPG that in turn projects to pelvic organs (Nadelhaft and Booth, 1984; Keast, 1995); (2) sensory axons that originate from sacral dorsal root ganglia and traverse the MPG on the way to the pelvic organs (Dail *et al*. 1975; Nadelhaft and Booth, 1984; Dail and Dziurzynski, 1985; Papka and McNeill, 1992; Dail, 1996); and (3) sympathetic postganglionic axons originating from the lumbar sympathetic chain (Alm and Elmer, 1975; Kuo *et al*. 1984; Dail *et al*. 1986). In rats, the pelvic nerve has been described as comprising three to seven distinct fascicles (Hulsebosch and Coggeshall, 1982; Arellano *et al*. 2019), loosely held together with delicate connective tissue. It is not known if these fascicles are functionally equivalent, either in their composition of sensory, preganglionic and sympathetic axons, or the organs that they control. Therefore, the initial specific goals of the present study were to first define the number and size of the pelvic nerve fascicles, then determine the primary components of each fascicle. Our approach was to visualise the projections of organ‐specific sensory axons labelled by microinjection of neural tracer (cholera toxin, subunit B) into the bladder, urethra or rectum. We also examined the location of the total population of peptidergic sensory axons within the pelvic nerves to determine whether their fascicular distribution was distinct from parasympathetic preganglionic and sympathetic postganglionic axons.

In a second component of the present study, we aimed to investigate the trajectory of sensory axons projecting in the pelvic nerve, from their point of entry to the MPG. This ganglion innervates all of the pelvic organs and is a mixed sympathetic‐parasympathetic ganglion, comprising autonomic ganglion cells regulated by preganglionic neurons in either the L1‐L2 spinal cord (sympathetic pathways) or the L6‐S1 spinal cord (parasympathetic pathways; Nadelhaft and Booth, 1984; Keast, 1999b). Utilising markers of the peptidergic class, sensory axons have previously been investigated within the MPG where they showed close associations with some neurons (Senba and Tohyama, 1988; Papka and McNeill, 1992). In the present study, we extend understanding of this potentially important site of crosstalk between sensory and autonomic systems by examining the projections and associations of axons immunoreactive for calcitonin gene‐related peptide (CGRP) in full‐thickness MPG with attached nerves.

## 2 Materials and methods

### 2.1 Animals

Procedures were conducted according to the institutional and funding body requirements for animal experimentation, approved by the Animal Ethics Committee of the University of Melbourne, and in compliance with the Australian Code for the Care and Use of Animals for Scientific Purposes (National Health and Medical Research Council of Australia). Male Sprague–Dawley rats (8–10 weeks old) were sourced from the Biomedical Sciences Animal Facility (University of Melbourne) and housed under a 12‐h light/dark cycle, in a temperature‐controlled room with *ad libitum* access to food and water. To reduce the use of animals in experimentation, in the majority of cases the ganglia and nerves were dissected from animals being used for other experiments (e.g. studies of spinal cord or organs) to be reported elsewhere.

### 2.2 Neural tracing

In this study, a neural tracer, cholera toxin, subunit B (CTB), was injected into either the bladder body, bladder trigone, urethra or rectum in order to label sensory and autonomic neurons that innervate these regions. General anaesthesia was induced in animals using isoflurane (3% in oxygen for induction and 1.5%–2% for maintenance of anaesthesia). Pre‐emptive analgesia was induced by subcutaneous injection of 0.05 mg/kg buprenorphine (Temgesic; Reckitt Benckiser, Hull, UK). A midline incision was made in the lower abdomen and overlying organs displaced. CTB (0.3% w/v in sterile water; List Biolabs, Campbell, CA, USA) was microinjected using a Neuros 5‐µL syringe equipped with a 33‐G needle (65460‐03; Hamilton Co., Reno, NV, USA). The total volume injected for each organ and number of injection sites were: bladder body 3–5 µL, six sites; bladder trigone (dorsal bladder neck, in the midline, at the level of ureter entry) 3–5 µL, two sites; proximal dorsal urethra (~1–2 mm caudal to the trigone, near the most rostral edge of the rhabdosphincter) 3–4 µL, two sites; and rectum (caudal to the inferior mesenteric artery) 4–5 µL, two sites. The incision was then closed using sutures and surgical clips, and animals were provided with postoperative analgesia via subcutaneous injection of buprenorphine (Temgesic: 0.05 mg/kg) within 10–12 h of surgery. The detailed protocol for tracer microinjection has been published previously (Keast and Osborne, 2019a).

In pilot studies, several transport times (4, 7, 14 days) were compared in order to optimise the visualisation of CTB‐labelled axons in the pelvic nerve following lower urinary tract (LUT) injection. As described in more detail in the Results section, the optimal transport time was 4 days, with some CTB axons visible at 7 days but very few CTB‐labelled axons visible 14 days after injection (four rats). All of the CTB data described below were obtained after 4–7 days of transport (bladder body, five rats; bladder trigone, five rats; urethra, seven rats; rectum, four rats). Pelvic ganglia and pelvic nerves from these animals were also used for mapping the distribution of immunohistochemically defined classes of axons.

Following the chosen transport period (see above), animals were anaesthetised (100 mg/kg ketamine and 10 mg/kg xylazine, i.p.) then perfused transcardially with saline (0.9% sodium chloride containing 1% sodium nitrate and 5000 IU/mL heparin), and freshly made fixative (4% paraformaldehyde in 0.1 M phosphate buffer, pH 7.4). Relevant organs were removed to confirm the location of the injection site(s) and MPGs (with associated nerves) dissected (Bertrand and Keast, 2020). During post‐fixation (1 h), the MPGs were secured with micropins to a dish lined with silicon polymer, to retain the shape and orientation of their major components. In the initial studies, several dorsal root ganglia (L6 and S1 spinal level) were also removed and post‐fixed (1 h) to demonstrate successful tracer injection and sufficient post‐surgical transport time. Tissues were then washed in phosphate‐buffered saline (PBS; 0.1 M, pH 7.2) and stored at 4°C in PBS containing 0.1% sodium azide until processed for immunohistochemistry. MPGs with their associated nerves were also removed from 10 naïve rats (no neural tracer) using the same perfusion and tissue processing procedures. The detailed protocol for intracardiac perfusion with fixative has been published previously (Keast and Osborne, 2019b).

### 2.3 Whole‐mount immunostaining protocol

The MPGs were washed in PBS (3 × 30 min), then incubated in blocking solution (PBS containing 0.5% Triton X‐100 and 10% horse serum (Merck; Darmstadt, Germany) for 2 h at room temperature. MPGs were then incubated with combinations of primary antibodies (Table 1) for 72 h at room temperature. The rationale behind the selection of these combinations is outlined in the Results section. MPGs were then washed in PBS (3 × 30 min) and incubated with combinations of species‐specific secondary antibodies (Table 2) for 18–24 h at room temperature, then washed in PBS (3 × 30 min), mounted on glass slides and cover‐slipped using Vectashield mounting medium (Vector Laboratories). Antibodies were diluted with PBS containing 0.1% sodium azide, 0.5% Triton X‐100 and 2% horse serum. Incubations and washes were carried out at room temperature on an orbital shaker.

**Table 1:** Primary antibodies

Antigen	Host species	Working dilution	Catalogue number	Manufacturer	RRID
CGRP	Goat	1:2000	1720‐9007	Bio‐Rad (AbD Serotec); Oxford, UK	AB_2290729
CGRP	Rabbit	1:5000	C8198	Merck (Sigma‐Aldrich); Darmstadt, Germany	AB_259091
CGRP	Mouse	1:2000	AB 81887	Abcam; Cambridge, UK	AB_1658411
ChAT	Goat	1:500	AB144P	Merck (Millipore); Darmstadt, Germany	AB_11214092
CTB	Rabbit	1:150 000	C3062	Merck (Sigma‐Aldrich)	AB_258833
CTB	Goat	1:10 000	703	List Biological Laboratories; Campbell, CA, USA	AB_10013220
PGP	Rabbit	1:2000	AB5925	Merck (Sigma‐Aldrich)	AB_11214054
SP	Rabbit	1:5000	20,064	Immunostar; Hudson, WI, USA	AB_572266
TH	Mouse	1:1000	22,941	Immunostar	AB_572268
TH	Sheep	1:1000	AB1542	Merck (Millipore)	AB_90755
VAChT	Rabbit	1:2000	139,103	Synaptic Systems; Goettingen, Germany	AB_2247684

Abbreviations: CGRP, calcitonin gene‐related peptide; ChAT, choline acetyltransferase; CTB, cholera toxin, subunit B; PGP, protein gene product 9.5; SP, substance P; Syn, synaptophysin; TH, tyrosine hydroxylase; VAChT, vesicular acetylcholine transporter.

**Table 2:** Secondary antibodies

Antigen	Conjugate	Working dilution	Catalogue number	Manufacturer	RRID
Goat IgG	Cy3	1:1000	705‐165‐147	Jackson ImmunoResearch Laboratories; West Grove, PA, USA	AB_2307351
Mouse IgG	AF488	1:1000	A21202	Thermo Fisher Scientific (Molecular Probes); Waltham, MA, USA	AB_141607
Mouse IgG	Cy3	1:2000	715‐165‐150	Jackson ImmunoResearch Laboratories	AB_2340813
Mouse IgG	AF647	1:200	A31571	Thermo Fisher Scientific (Molecular Probes)	AB_162542
Rabbit IgG	Cy3	1:3000	711‐165‐152	Jackson ImmunoResearch Laboratories	AB_2307443
Rabbit IgG	AF647	1:1000	A21207	Thermo Fisher Scientific (Molecular Probes)	AB_141637
Rabbit IgG	AF488	1:1000	711‐545‐152	Jackson ImmunoResearch Laboratories	AB_2313584
Sheep IgG	AF647	1:500	A21448	Thermo Fisher Scientific (Molecular Probes)	AB_2535865

### 2.4 Microscopic analysis

All MPGs were first assessed using wide‐field fluorescence microscopy (Zeiss AxioImager M2, Zeiss AxioImager Z1) and selected regions were then analysed using confocal microscopy (Zeiss LSM800, LSM880). Ganglia were only included in the study if the body of the MPG (i.e. the primary aggregation of neuronal cell bodies) and the location of the pelvic and cavernous nerves could be identified. We assessed the pelvic nerves in all intact whole‐mount preparations from transcardially perfused naïve and CTB‐injected animals as follows:

- Fascicles were numbered, commencing at the fascicle closest to the entry of the cavernous nerve to the MPG. These numbers were systematically used to reference all descriptions of CTB‐labelled axons or immunohistochemically classified axon types.
- The diameter of each fascicle was measured 300 μm from its entry point into the MPG.
- Where a sufficient length of pelvic nerve was collected, fascicles were assessed to the point of junction with the levator ani nerve.
- An initial assessment of the topographical organisation of different types of axons within fascicles was made, and subsequently validated by confocal microscopy (40× or 63× oil immersion objectives; orthogonal views of z‐stacks). This included axons immunolabelled for CTB and/or immunohistochemical markers of different functional classes.
- Observations were made on the trajectory of sensory axons from their point of entry to the MPG.

### 2.5 Figure production and data availability

Figures were produced using Adobe Creative Suite (InDesign, Photoshop; Adobe Systems). Monochrome images, captured using Zen software (Zeiss), were colourised to optimally distinguish the structures of interest, and adjustments were made to brightness and contrast to best illustrate the signal as visualised directly with the microscope. To facilitate comparison across figures, images of pelvic nerve have been oriented consistently with fascicles 1–2 on the left and 3–5 on the right, with the top of the image being closest to the MPG. The fascicles of the pelvic nerve are held together quite loosely, so were frequently separated during the mounting and cover‐slipping processes.

Data that support the findings of the study will be publicly available at the National Institute of Health‐supported SPARC public portal, sparc.science (SPARC Project RRID:SCR_017041).

## 3 Results

### 3.1 Location and mesoscopic features of the pelvic nerve

**Fig. 1:**
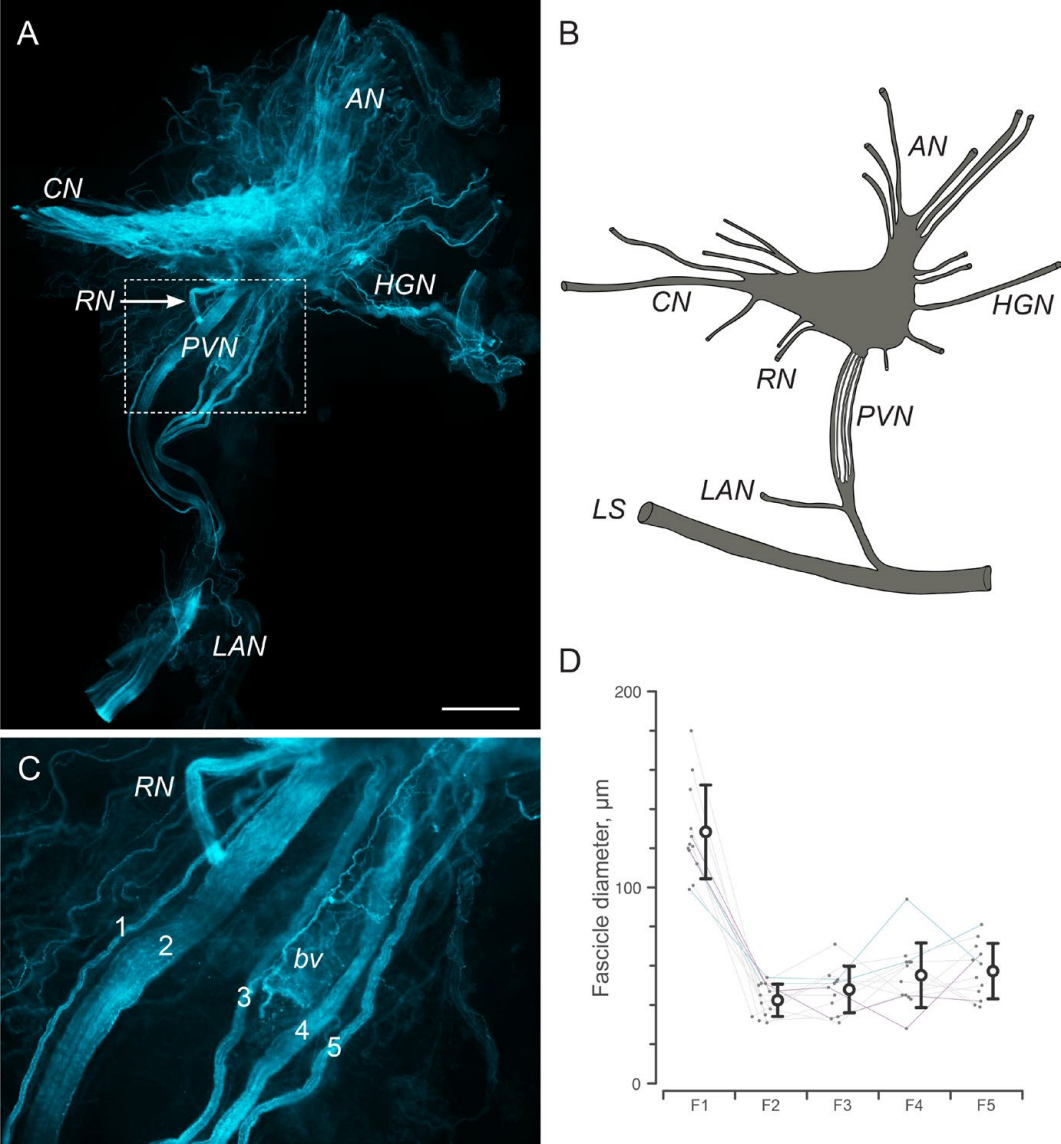
Location and fascicle structure of the male rat pelvic nerve. (A) Major pelvic ganglion (MPG; left side of the rat) with associated nerves, including the pelvic nerve (PVN), hypogastric nerve (HGN), accessory nerves (AN) and cavernous nerve (CN). One of the rectal nerves (RN) is still attached. The dissected ganglion has been immunostained for calcitonin gene‐related peptide, so shows the extensive distribution of sensory axons in many of the nerves and throughout the MPG. Higher magnification of the region where the PVN enters the MPG is shown in panel C. (B) Schematic showing the primary features of the MPG and its associated nerves as they would appear prior to dissection, with its longer connections intact, including the levator ani nerve (LAN) and L6‐S1 trunk (LS). (C) Five distinct fascicles of the PVN are evident, labelled here from 1 to 5, commencing from the side nearest the cavernous nerve. A blood vessel (bv) travelling with the PVN is also retained in the dissection. Calibration bar in panel A represents: 1 mm (A) and 150 µm in panel C. (D) Diameter of PVN fascicles from nine rats. Each grey line indicates a single PVN measured from one rat. In two rats, both PVNs were measured; each of these rats is indicated by a different colour. Post‐dissection, it was sometimes difficult to determine which of fascicles 1 and 2 originated closer to the cavernous nerve, so for the purpose of overall comparison, the largest of the two fascicles was designated fascicle 1. [Colour figure can be viewed at wileyonlinelibrary.com]

The pelvic nerve is one of the major terminal branches of the L6‐S1 spinal nerve trunk. Our priority during dissection was to retain the junction of the pelvic nerve with the MPG to provide a point of reference sustainable during the subsequent processes of tissue processing and analysis. The pelvic nerve joins the MPG on its dorsal aspect, between the cavernous and hypogastric nerves (Fig. 1A,B). The accessory nerves, comprising several clusters of fine nerves projecting to the reproductive and urinary tracts, exit from the most ventral aspect of the MPG. In many cases, small lengths of rectal nerves also remained attached to the dissected MPG (Fig. 1A–C). In some dissections, we removed the entire length of pelvic nerve to its junction with the levator ani nerve (Fig. 1A,B). After fixation, this length was 5682 ± 306 μm (*n* = 6, measuring one pelvic nerve for each of six rats).

Distinct fascicles of the pelvic nerve were clearly evident during dissection and often became separated during dissection and subsequent tissue handling. The fascicles remained quite distinct from each other along the length of the pelvic nerve to the junction with the levator ani nerves, where they then merged. We quantified the number of fascicles in 19 pelvic nerves taken from 13 animals. All pelvic nerves comprised five distinct fascicles, with the exception of two pelvic nerves that each had only four fascicles. Delicate connective tissue associated with these fascicles contained several very small bundles of axons that were only a few micrometers in diameter. These axons appeared to be primarily associated with microvasculature and were not included in our classification of fascicles. A larger blood vessel (>100 µm diameter), clearly evident in dissection, was typically embedded amongst the pelvic nerve fascicles and often retained during dissection. This vessel continued to follow the edge of the MPG or penetrated the middle of the ganglion to travel along the surface of the prostate gland.

In 12 of these nerves (from nine animals) that had five fascicles, we could distinguish a consistent pattern of aggregation at their entry point to the MPG, enabling us to specifically label and measure the diameter of each fascicle (Fig. 1C,D). We numbered the fascicles 1 to 5, commencing at the site nearest the cavernous nerve. The first two fascicles always comprised a large (≥100 µm) and a small (<55 µm) fascicle. It was not always clear or consistent which of these two joined the MPG closest to the cavernous nerve, but this pair of fascicles was consistently separated from the other three (fascicles 3–5). This latter group of three fascicles varied in size but were all smaller than the larger of fascicles 1 and 2. The total diameter of the five fascicles was similar across individual animals (mean ± SD: 331 ± 27 µm; *n* = 12). In three animals the measurements were made on both left and right pelvic nerves and showed a high level of similarity (361 vs. 365, 322 vs. 325 and 290 vs. 316 µm).

### 3.2 Visualisation of peripheral neural pathways projecting to the lower urinary tract

**Fig. 2:**
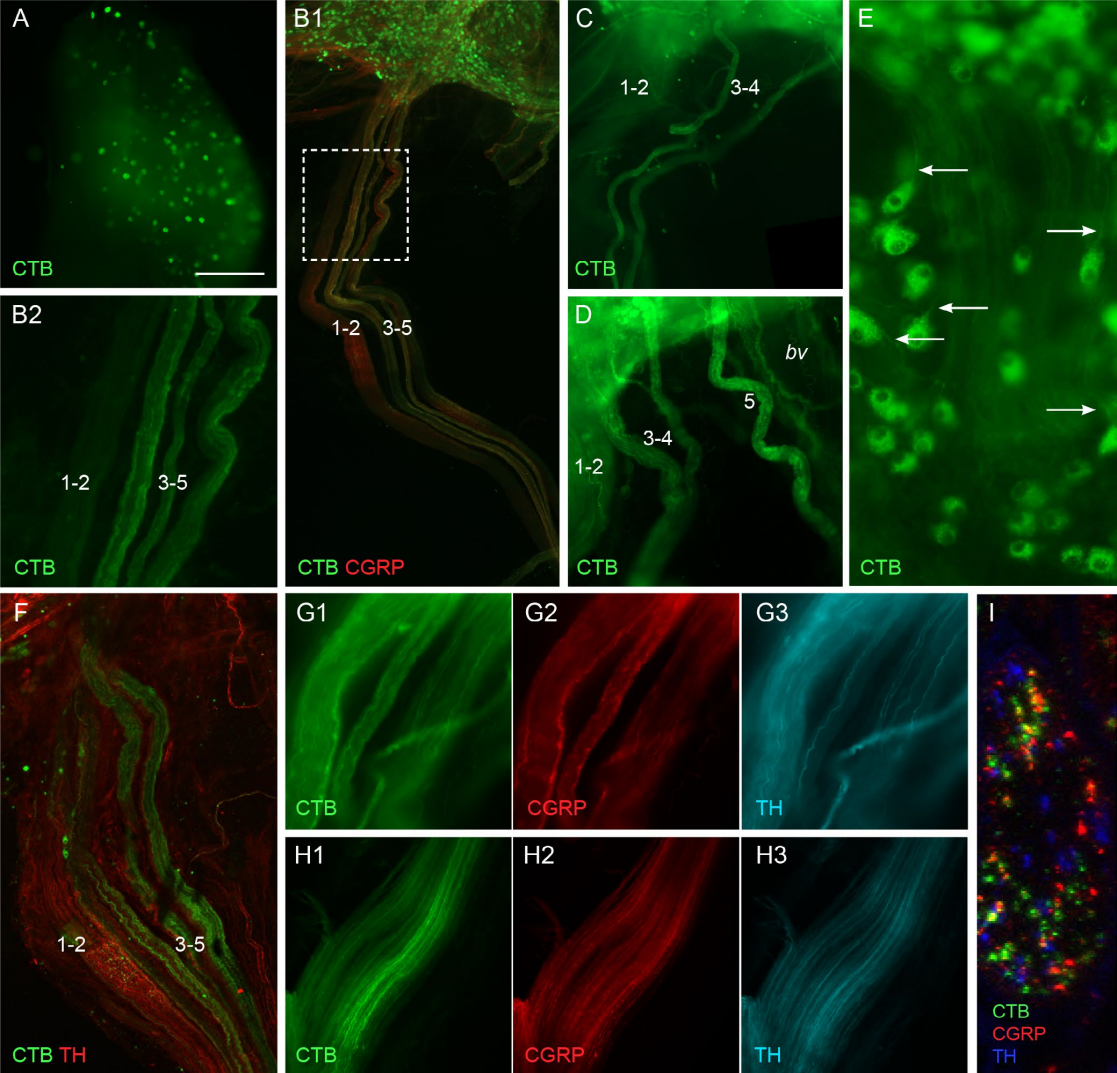
Visualisation of sensory and autonomic neural projections to the lower urinary tract labelled following cholera toxin, subunit B (CTB) injection into the bladder body (C, H, I), bladder trigone (A, D) or proximal urethra (B, E, F, G). (A) L6 dorsal root ganglion, showing numerous CTB‐labelled neurons. (B) In B1, the pelvic nerve is shown at low magnification, with part of the major pelvic ganglion (MPG) at the top of the panel. Many MPG neurons are labelled with CTB. The pelvic nerve is shown at higher magnification in B2. The five fascicles of the pelvic nerve are numbered, with the smaller of fascicles 1 and 2 obscured by the larger fascicle. The majority of CTB‐positive axons are located in fascicles 3 to 5. Peptidergic calcitonin gene‐related peptide (CGRP) axons are located in all fascicles. (C, D) CTB‐positive axons aggregated in pelvic nerve fascicles 3–5 (fascicle 5 not shown in C). (E) CTB‐positive autonomic ganglion neurons in the MPG, showing bright immunoreactivity in cell bodies and faint labelling of proximal axons (arrows). (F) Pelvic nerve showing aggregation of CTB‐positive axons in fascicles 3–5 and their site of entry to the MPG (top); co‐staining for tyrosine hydroxylase (TH) shows the location of fascicles 1 and 2. (G) and (H) provide higher magnification views of fascicles 3–5 close to the MPG (G) and ~ 1 mm along the pelvic nerve (H); sensory and sympathetic axons are present in each fascicle. (I) Transverse section through a pelvic nerve fascicle containing many CTB‐positive axons, showing that many are CGRP‐positive but none are TH‐positive. A–E, G, H: wide‐field fluorescence microscopy; F, I, confocal microscopy. Calibration bar in panel a represents (µm): A (500), B1 (500); B2 (160); C (400), D (200), E (60), F (180), G and H (60), I (10). [Colour figure can be viewed at wileyonlinelibrary.com]

We did not detect any difference in the location or features of the CTB labelling within ganglia or the pelvic nerve after injection of the bladder body, bladder trigone or proximal urethra, so have aggregated these observations. Examples from each region are provided in Fig. 2.

Cholera toxin, subunit B (CTB)‐positive neuronal cell bodies were identified in L6‐S1 dorsal root ganglia (Fig. 2A) and MPG (Fig. 2B,E), concurring with previous studies utilising fluorescent retrograde tracers, Fast Blue and FluoroGold (Nadelhaft and Booth, 1984; Keast *et al*. 1989; Keast and de Groat, 1992). CTB‐positive neurons were distributed widely across the MPG. CTB labelling was visible within axons of the pelvic nerve only at shorter transport times of 4 or 7 days and was most clearly observed at 4 days (Fig. 2B–D, F–I). CTB‐positive axons were consistently located within fascicles 3–5 but sparse or absent in fascicles 1 or 2 (Fig. 2B,C,F). Across fascicles 3–5, CTB‐positive axons generally showed some variation in axon density and distribution; however, we were unable to detect a consistent pattern, e.g. to reflect a higher or lower density or distribution of CTB‐positive axons within a particular fascicle. The aggregation of CTB‐positive axons in fascicles 3–5 continued along the length of the pelvic nerve (Fig. 2G1, H1) to its junction with the levator ani nerve. From the point where the pelvic nerve merged with the MPG, it was generally difficult to visualise CTB‐labelled axons; however, CTB axons could be seen within the nerve tracts (accessory nerves) that project from the MPG to the LUT (not shown). No CTB‐positive axons were detected in the cavernous or rectal nerves. Some lightly labelled axons emerged from CTB‐positive MPG neurons (Fig. 2E); these autonomic axons may also contribute to the CTB axons seen in the accessory nerves.

We also took this opportunity to visualise peptidergic sensory and noradrenergic sympathetic axons in the pelvic nerve, by co‐staining for CGRP and tyrosine hydroxylase (TH), respectively (Fig. 2G–I). This demonstrated that both axon classes are found in all five fascicles, as examined in more detail below. Confocal microscopy showed that many but not all CTB‐positive axons were CGRP‐immunoreactive, but none showed TH immunoreactivity. We therefore deduced that CTB‐positive axons in the pelvic nerve represented both peptidergic and non‐peptidergic sensory projections to the LUT.

### 3.3 Visualisation of peripheral neural pathways projecting to the large intestine (rectum)

**Fig. 3:**
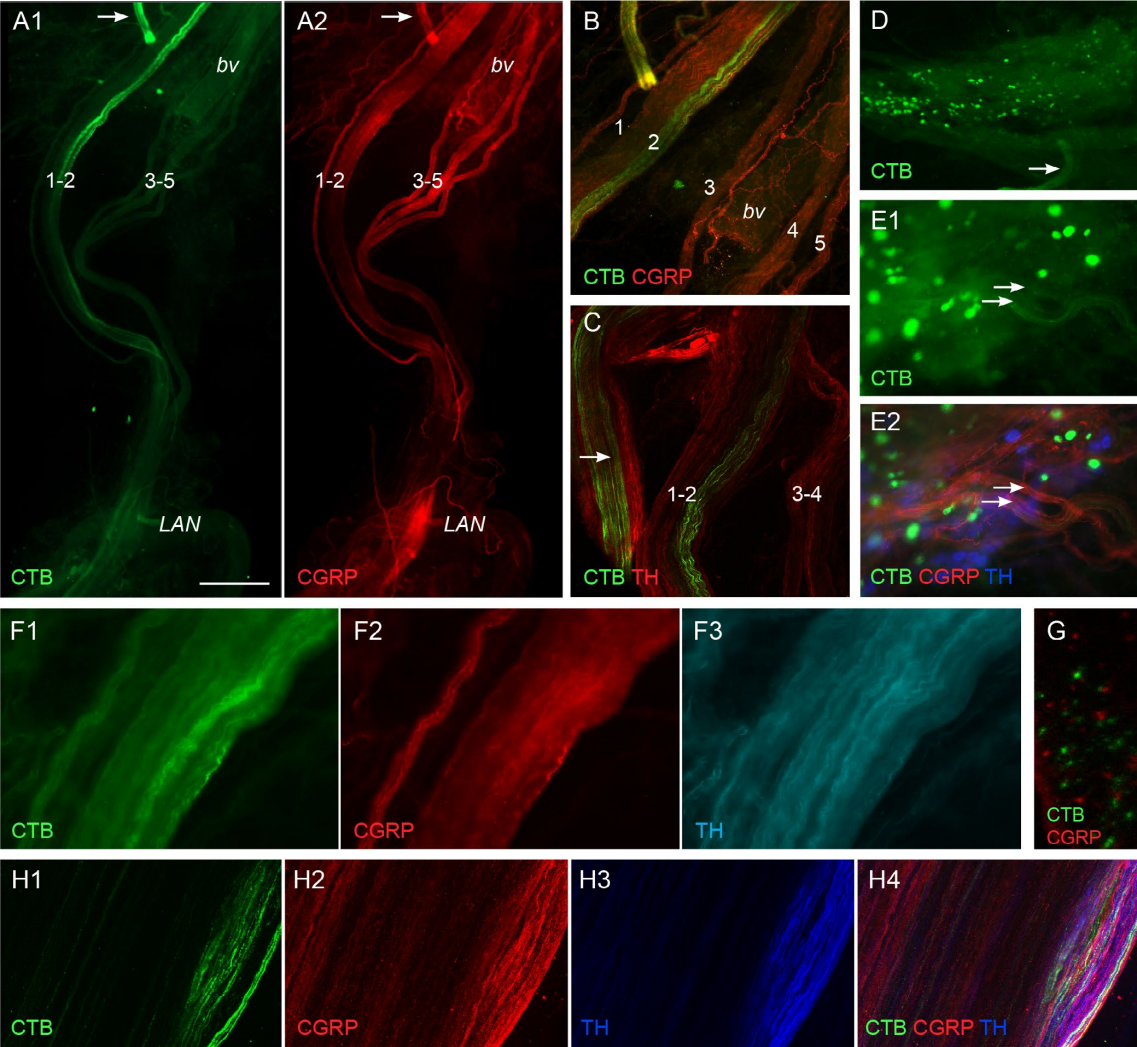
Visualisation of sensory and autonomic neural projections to the large intestine labelled following cholera toxin, subunit B (CTB) injection into the rectum. (A) CTB axons within the pelvic nerve are aggregated in fascicles 1 and 2. They maintain this aggregation along the length of the pelvic nerve, to the levator ani nerve (LAN). A blood vessel (bv) is closely associated with fascicles 3–5. Part of a rectal nerve is also shown at the top of the field (arrow). Peptidergic calcitonin gene‐related peptide (CGRP) axons project in all fascicles. (B) Image showing higher magnification of fascicles from panel A, with both channels merged. A and B are from the same ganglion shown in Fig. 1. (C) CTB‐positive axons aggregated in the rectal nerves (arrow) and in fascicles 1 and 2. These tracts also contain many sympathetic noradrenergic axons tyrosine hydroxylase (TH). (D) Major pelvic ganglion (MPG) with many ganglion neurons labelled by CTB. One of the rectal nerves is indicated (arrow). (E) Edge of the MPG near the rectal nerves, two of which are indicated by arrows. Both contain many CGRP‐positive axons; some CTB‐positive axons are also visible in the lower of the two nerves. (F) Higher magnification of fascicle 2, showing CTB‐positive axons and many axons immunolabelled for CGRP or TH. (G) Transverse section through a pelvic nerve fascicle containing CTB‐positive axons, this region showing many that are CGRP‐negative. (H) Fascicle 2 showing strongly aggregated CTB‐positive axons intermingled with axons immunolabelled for CGRP or TH. A region in the middle of the field shows no immunolabelling and is deduced to contain non‐peptidergic sensory axons and sacral (parasympathetic) preganglionic axons. A–F: wide‐field fluorescence microscopy; G, H: confocal microscopy. Calibration bar in panel A represents (µm): A (400), B (200), C (200), D (400), E (150), F (80), G (20) and H (80). [Colour figure can be viewed at wileyonlinelibrary.com]

The shorter transport time (4 days) was used for the group of studies examining the peripheral neural pathways projecting to the large intestine. CTB‐positive axons were consistently located within the pelvic nerve after injection of CTB into the rectal wall. These axons were aggregated strongly in the larger of fascicles 1 and 2 but were rarely observed in fascicles 3–5 (Fig. 3A–C). Similar to our observations following CTB injection into the LUT, from the point where the pelvic nerve merged with the MPG, it was generally difficult to visualise CTB‐labelled axons; however, CTB‐positive axons were detected in the rectal nerves (Fig. 3A–E) but not the cavernous or accessory nerves. CTB‐positive neuronal cell bodies were identified in L6‐S1 dorsal root ganglia (not shown) and MPG (Fig. 3D,E), concurring with previous studies using fluorescent tracers (Keast *et al*. 1989; Keast and de Groat, 1992). Within the MPG, CTB‐positive neurons were aggregated near the junction of the cavernous and rectal nerves, as previously reported (Keast *et al*. 1989; Luckensmeyer and Keast, 1995). Fewer MPG neurons were labelled after CTB injection into the rectum compared to any of the regions of the LUT. CTB labelling was difficult to identify in the axons emerging from rectum‐projecting MPG neurons.

Within the pelvic nerve, CTB‐positive axons were consistently detected within fascicles 1 and 2, with the majority located in the larger of these fascicles. Here, the CTB‐positive axons were strongly clustered in one region rather than distributed evenly across the fascicle. The aggregation of CTB‐positive axons in fascicles 1 and 2 continued along the length of the pelvic nerve to its junction with the levator ani nerve (Fig. 3A). Co‐staining for CTB with CGRP and TH, respectively, demonstrated again that both nerve types are found in all five fascicles, as examined in more detail in the following section. In fascicles 1 and 2, many but not all CTB‐positive axons were CGRP‐immunoreactive, but none showed TH immunoreactivity (Fig. 3F–H). We therefore deduced that CTB‐positive axons in the pelvic nerve represented both peptidergic and non‐peptidergic sensory projections to the rectum.

### 3.4 Distribution of sensory and autonomic axons in the pelvic nerve

**Fig. 4:**
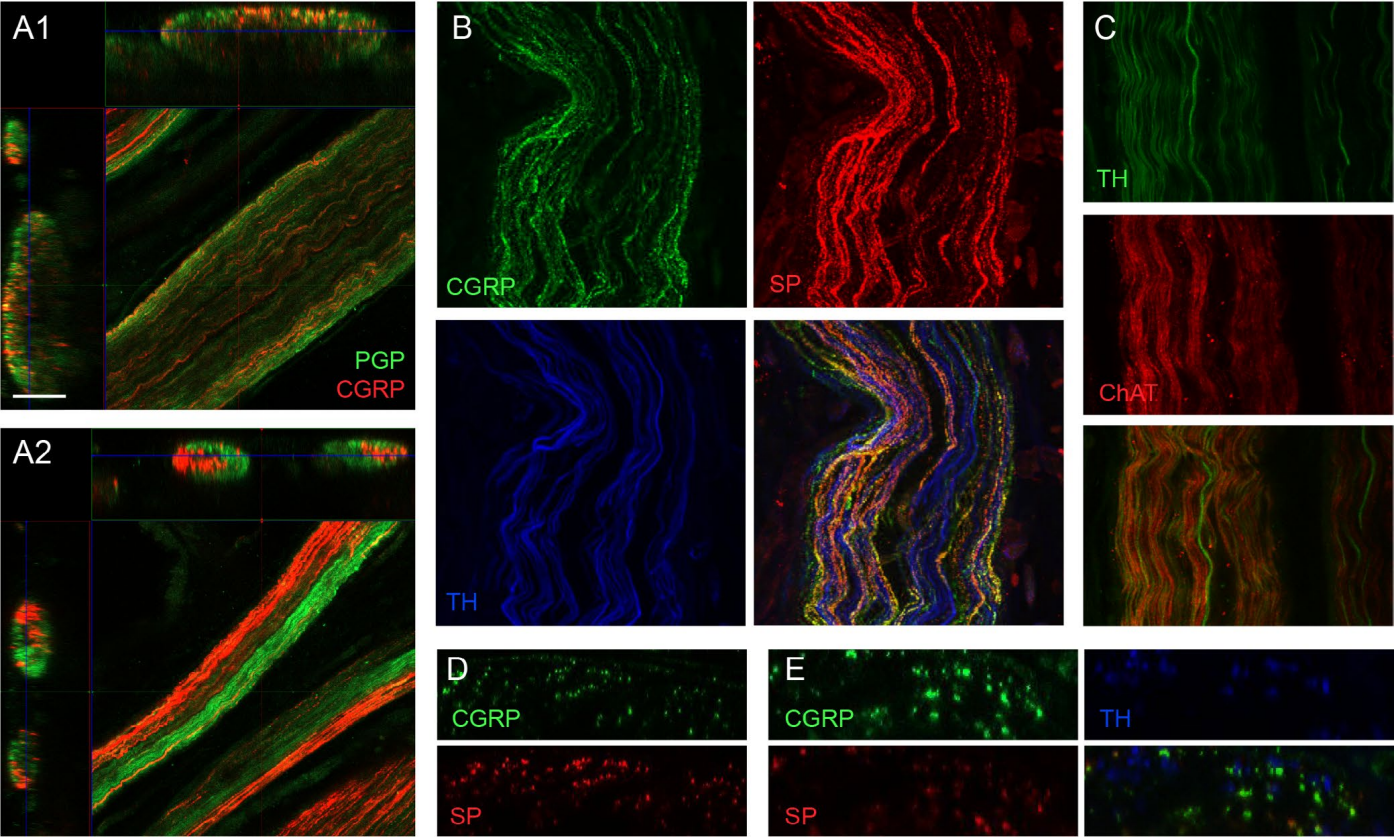
Distribution of sensory and autonomic axons in the pelvic nerve. Confocal micrographs oriented parallel or perpendicular to the long axis of the nerve. (A) Fascicles of the pelvic nerve showing axons labelled for calcitonin gene‐related peptide (CGRP) and the pan‐axonal marker, protein gene product 9.5 (PGP). The larger fascicle (A1, fascicle 1) shows relatively uniform distribution of CGRP axons, whereas in the smaller fascicles (A2, fascicles 2 and 3), CGRP‐positive axons are strongly aggregated. (B) Strong but not complete co‐expression of the two peptides, calcitonin gene‐related peptide (CGRP) and substance P (SP) in fascicle 1. Noradrenergic sympathetic postganglionic axons labelled for tyrosine hydroxylase (TH) are interspersed between the peptidergic sensory axons. (C) Noradrenergic sympathetic postganglionic axons (TH) are distinct from cholinergic axons labelled for choline acetyltransferase (ChAT), most or all of which are likely to be preganglionic axons. Here this is demonstrated in fascicles 2 and 3. (D) Strong but incomplete co‐expression between the two peptides, CGRP and SP, in fascicles 4 and 5. (E) In this region of fascicle 1, many of the CGRP axons are SP‐negative; noradrenergic axons (TH‐positive) do not express either peptide. Calibration bar in panel A represents (µm): A (50), B (30), D (30), D (25), E (10). [Colour figure can be viewed at wileyonlinelibrary.com]

We extended our analyses of axon classes in the pelvic nerve using confocal microscopy to assess pelvic nerves from animals that had not undergone tract tracing with CTB (Fig. 4). This enabled use of a greater number of antibodies to compare distribution of sensory and autonomic axon classes. We first examined in more detail the location of CGRP‐positive axons across the five fascicles, using a pan‐axonal marker, protein gene product 9.5 (PGP), to visualise the total axon population. We found that in most pelvic nerve specimens, CGRP‐positive axons were distributed quite evenly across the larger of the five fascicles, but in the smaller fascicles, CGRP‐positive axons were commonly clustered in one region of the fascicle (Fig. 4A). Many but not all of the CGRP‐positive axons were also immunoreactive for substance P (SP) (Fig. 4B,D,E). Axons immunolabelled for these neuropeptides typically showed punctate staining.

Sympathetic postganglionic axons immunolabelled for TH were found in all fascicles and frequently intermingled with the CGRP‐positive axons (Fig. 4B,D,E). TH‐positive axons were identified in all fascicles but were particularly prevalent in fascicles 1 and 2. Axons immunolabelled for TH typically showed smooth rather than punctate staining (Fig. 4B,C). No co‐expression was detected of TH with SP or CGRP. We also attempted to visualise cholinergic preganglionic axons in the pelvic nerve by immunolabelling for choline acetyltransferase (ChAT). While some signal was detected in each of the fascicles, the quality of labelling was generally poor and inconsistent across preparations. In only a few cases could we confidently determine co‐expression patterns (Fig. 4C). In these, we noted that ChAT did not co‐label with TH, SP or CGRP.

### 3.5 Projections of CGRP‐positive sensory axons within the MPG

To investigate the trajectory of sensory axons projecting in the pelvic nerve, we closely followed tracts of CGRP‐positive axons from their point of entry to the MPG, co‐staining selected ganglia for TH (to distinguish noradrenergic from cholinergic neurons) or CTB (for ganglia removed from animals undergoing CTB microinjection into the LUT or rectum).

**Fig. 5:**
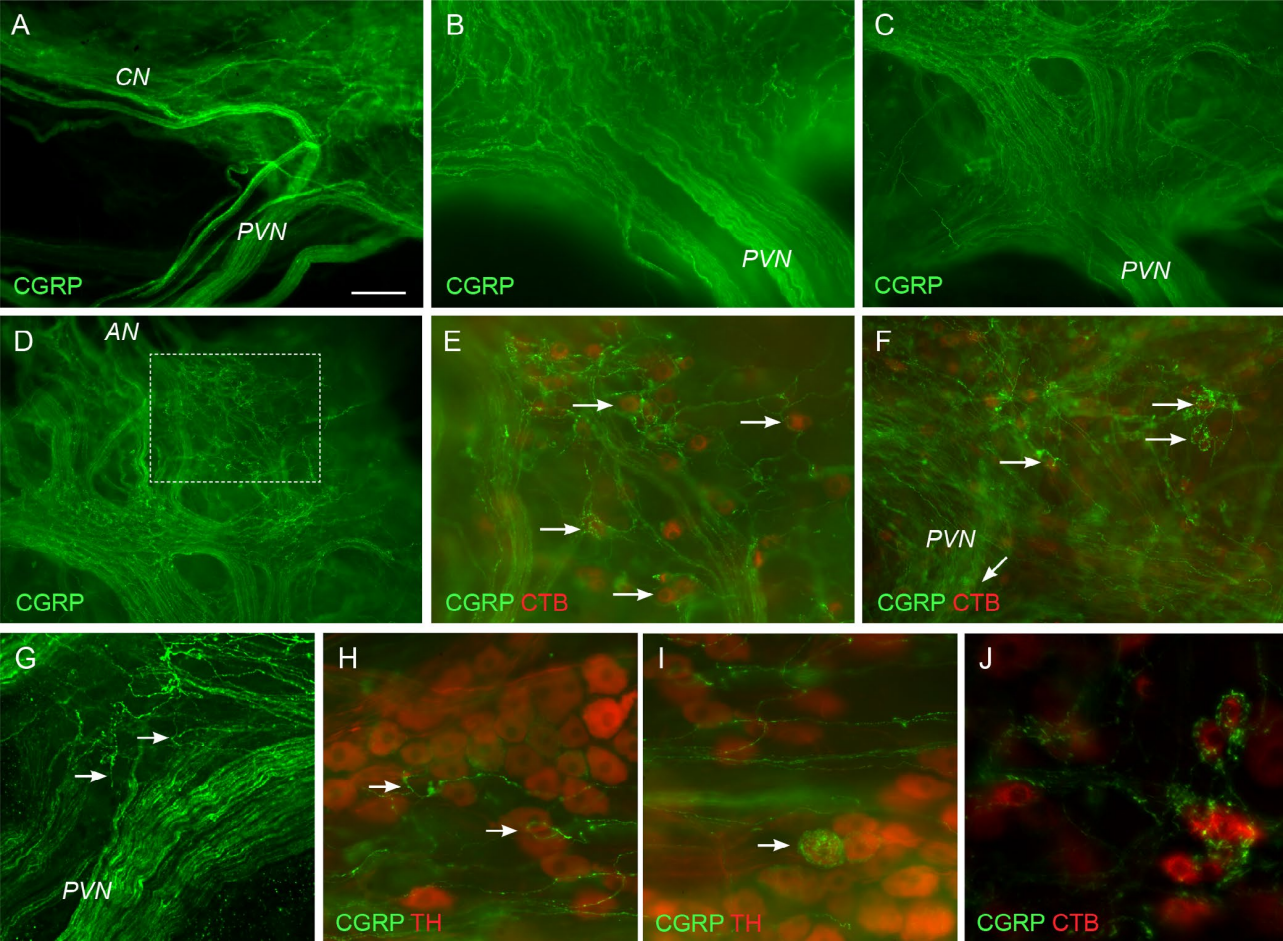
Projections of calcitonin gene‐related peptide (CGRP)‐positive axons in the major pelvic ganglion (MPG). Panels B–F and J are images from the same MPG. Red neurons in E, F and J are labelled from a cholera toxin, subunit B (CTB) injection into the urethra. Ganglia in A–G are oriented with the pelvic nerve towards the bottom and the cavernous nerve to the left. (A) From their point of entry to the MPG, many CGRP‐positive axons project towards the cavernous nerve (CN). (B) Dense tracts of CGRP‐positive axons within pelvic nerve (PVN) fascicles at their point of junction with the MPG. (C) Many CGRP‐positive axons in the pelvic nerve remain aggregated in tracts and cross the surface of the MPG. (D) Dense CGRP‐positive axon tracts traversing the MPG surface project to the most ventral edge, towards the accessory nerves (AN); higher magnification of outlined region is shown in (E), merged with the image of CTB‐labelled ganglion neurons. This depicts a region close to the exit of the accessory nerves where many CTB‐positive neurons are closely associated with CGRP‐positive axons; examples indicated by arrows. (F) Focusing more deeply within the ganglion near the junction with the pelvic nerve, many CGRP‐positive axons separate from the major tracts and surround individual neurons; examples indicated by arrows. (G) Examples (arrows) of individual varicose axons emerging from the pelvic nerve, to travel within the ganglion tissue. (H) Varicose CGRP‐positive axons encircle two tyrosine‐hydroxylase (TH)‐negative neurons (arrows). (I) At the top of the field a loose network of delicate, varicose CGRP‐positive axons is located between tyrosine hydroxylase (TH)‐positive ganglion neurons but does not encircle them. In the lower part of the field, one of the TH neurons (arrow) is surrounded by numerous CGRP‐positive varicosities, but the axon providing these varicosities is CGRP‐negative. (J) Varicose CGRP‐positive axons surround an aggregate of CTB‐positive neurons (urethra injection). All images taken using wide‐field fluorescence microscopy. Calibration bar in panel A represents (µm): A (200), B (75), C (150), D (150), E (75), F (100), G (75), H, I (50), J (40). [Colour figure can be viewed at wileyonlinelibrary.com]

Upon joining the MPG, many CGRP‐positive axons traversed the surface of the ganglion to project along the cavernous nerve (Fig. 5A,B). Others traversed the surface to either exit in the rectal nerves (Fig. 5A,D,E) or were directed towards the accessory nerves (Fig. 5C,D). Tracts projecting to the cavernous and rectal nerves primarily arose from fascicles 1 and 2, whereas those projecting more ventrally, towards the accessory nerves, mainly originated from fascicles 3–5. The large tracts of CGRP‐positive axons directly traversing the ganglion were located on the outermost layer of the ganglion, on the surface facing the pelvic wall, rather than intermingling with ganglion neurons.

We also observed many CGRP‐positive axons closely associated with MPG neuron somata. These axons were of two types. One type could be followed from CGRP‐positive varicose axons that meandered through the ganglion tissue and then either partly or entirely encircled individual neurons or small groups of neurons (Fig. 5E–H; top of Fig. 5I). These axons showed CGRP labelling in their varicosities and inter‐varicose segments. They were associated only with TH‐negative (i.e. cholinergic) MPG neurons (Fig. 5H). Analysis of MPGs from CTB tracing studies showed that many of these CGRP‐positive axons were associated with MPG neurons that innervate the LUT (Fig. 5J), but none were identified to be associated with MPG neurons that innervate the rectum.

A second type of CGRP‐positive neuronal structure comprised dense aggregates of varicosities encapsulating a specific subpopulation of MPG neurons (Fig. 5I). CGRP could not be detected in the axon giving rise to these varicosities or the inter‐varicose segment of the axon. These varicosities were associated only with TH‐positive neurons and none were identified with CTB‐positive neurons innervating the LUT or the rectum.

### 3.6 Characterisation of CGRP axon terminations within the major pelvic ganglion

It has been reported that CGRP‐positive axons form terminations within the MPG (Senba and Tohyama, 1988; Papka and McNeill, 1992; Eastham *et al*. 2015) but have undergone limited characterisation. These, along with SP‐positive axons within MPGs (Dail and Dziurzynski, 1985), have generally been considered collaterals of sensory axons passing through the MPG to innervate pelvic organs. We identified no CGRP‐ or SP‐positive cell bodies in the MPG.

**Fig. 6:**
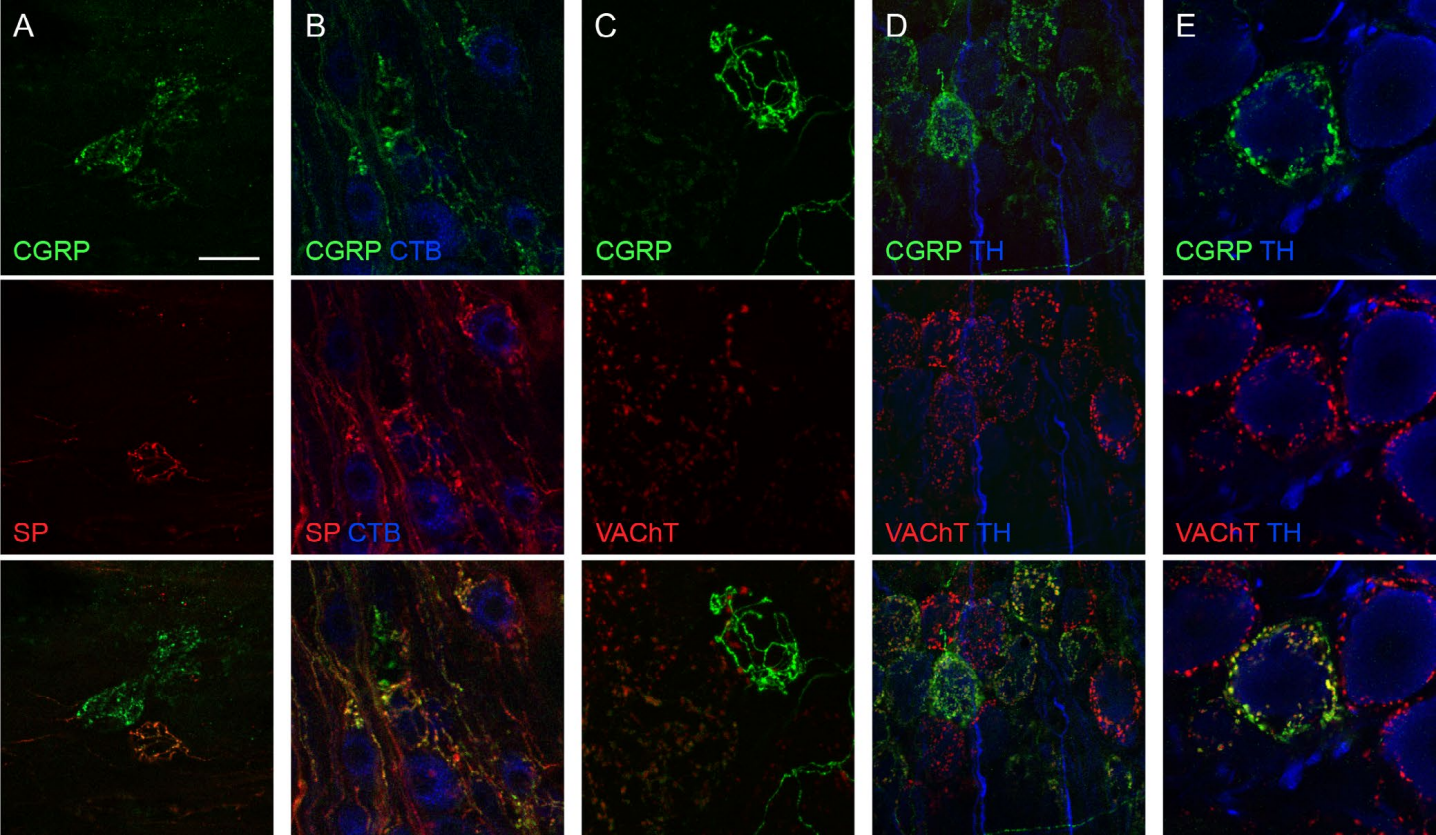
Features of two main classes of calcitonin gene‐related peptide (CGRP)‐positive axons in the major pelvic ganglion (MPG). (A) CGRP‐positive axons surrounding several MPG neurons. Some neurons are associated with dense CGRP‐positive, substance P (SP)‐negative varicosities, but one neuron is surrounded by an axonal plexus that is both CGRP‐ and SP‐positive. (B) Cholera toxin, subunit B (CTB)‐positive neurons retrogradely labelled from the bladder body. Some of these CTB neurons are associated with varicose axons that are SP‐ or CGRP‐positive, or labelled for both peptides. (C) Varicose CGRP axon encircling an MPG neuron, showing CGRP in the varicosities and the inter‐varicose segments of the axon. This varicose plexus is vesicular acetycholine transporter (VAChT)‐negative, but the neuron is also supplied by VAChT‐positive, CGRP‐negative terminals. Another CGRP‐positive, VAChT‐negative plexus of axons is at the bottom right of the image, and on the left, a field of neurons with VAChT‐positive but weak or absent CGRP labelling. (D) A region of MPG where many of the neurons are supplied by boutons immunoreactive for VAChT‐positive boutons, with or without CGRP immunoreactivity. (E) Higher magnification of noradrenergic neuron supplied by boutons that show both CGRP‐ and VAChT‐immunoreactivity, amongst other noradrenergic neurons with no nearby CGRP‐positive boutons. Calibration in panel A represents (µm): A (40), B (30), C (20), D (40), E (20). [Colour figure can be viewed at wileyonlinelibrary.com]

We first examined the CGRP‐positive varicose axons that were closely associated with many cholinergic MPG neurons, including neurons that innervate the LUT but not the rectum. By performing double‐labelling with antibodies against SP and CGRP, we found a strong but incomplete colocalisation of the two peptides in these structures (Fig. 6A,B). We then confirmed that these are likely to be sensory rather than autonomic preganglionic axons by immunolabelling for vesicular acetycholine transporter (VAChT). None of these SP‐ or CGRP‐positive axons were VAChT‐positive, although MPG neurons associated with these axon structures were supplied by VAChT‐positive varicosities (Fig. 6C).

The second type of CGRP‐positive axons comprised dense varicosities associated with a subpopulation of noradrenergic MPG neurons. These varicosities varied in their intensity of CGRP immunolabelling, did not co‐label for SP but were always labelled for VAChT (Fig. 6D,E). We deduced that these CGRP‐positive axons represent a specific subpopulation of sympathetic preganglionic axon terminals. Noradrenergic neurons supplied by these CGRP‐ and VAChT‐positive terminals did not have a unique location within the MPG and were intermingled with noradrenergic neurons innervated by CGRP‐negative, VAChT‐positive terminals.

## 4 Discussion

The focus of the present study was the microstructure of the pelvic nerve, a major peripheral nerve that carries most of the sensory and motor innervation to the pelvic organs. The diversity and breadth of this neural input, and the nature of its primary components, provide a strong parallel to the vagus nerve that carries a similarly extensive range of neural pathways to more rostral organs. To our knowledge, this is the first study to identify functionally relevant structural specialisations within regions of the pelvic nerve. This more advanced understanding of nerve organisation forms a foundation for future studies directed to modulation of particular neural classes or organ effects. Our related analyses of CGRP axons associated with the MPG provide further insights into the potential mechanisms by which sensory modulation may influence LUT function.

The pelvic nerve is considered a single nerve but has distinct components (fascicles) that are very loosely bound together rather than having a defined epineurium. A previous ultrastructural study of the pelvic nerve in male rats (Hulsebosch and Coggeshall, 1982) noted the existence of 5–7 fascicles, although we identified five fascicles in most animals, with four fascicles seen in a minority of cases. These fascicles are visible during dissection, although several much smaller axon tracts also exist. It is possible that the more delicate structures were classified as fascicles in the previous ultrastructural study. We found that the total diameter of fascicles was remarkably consistent within and between animals. We also identified a consistent set of features of the two fascicles closest to the cavernous nerve that were distinct from features of the other three fascicles. However, we were unable to further distinguish or consistently name the fascicles within these aggregates (1–2, 3–5) because the three‐dimensional structure of the nerve could not be maintained during tissue dissection and processing. Large‐volume imaging approaches to visualise the pelvic nerve still attached to the MPG and the spinal nerves may facilitate this further distinction. This approach may also enable tracing of axons that accompany the microvasculature, which were not specifically traced in the present study. It is important to repeat this study in female rats, where the primary components of the pelvic nerve are similar, but the number of axons is expected to be much lower. This sexual dimorphism is predicted on the basis of the smaller number of neurons in the female rat MPG (Purinton *et al*. 1973; Greenwood *et al*. 1985) that is innervated by spinal preganglionic neurons projecting in the pelvic nerve. It is also possible that the sensory and sympathetic components of the pelvic nerve are sexually dimorphic (McLachlan, 1985; Janig and McLachlan, 1987; Smith‐Anttila *et al*. 2020).

Microinjection of CTB enabled us to visualise the peripheral axons of lumbosacral sensory neurons that innervate the lower urinary or digestive tracts. This tracer binds to the GM1 ganglioside that is present in many axon terminals and has been widely used in studies of neural circuitry (Wu *et al*. 1999; Christianson *et al*. 2006; Christianson *et al*. 2007; Shehab and Hughes, 2011). Previous studies of primary sensory neurons in dorsal root ganglia have focused on somatic rather than visceral afferents and demonstrated a preferential labelling of the myelinated class (Shehab and Hughes, 2011), however CTB labels both myelinated and unmyelinated visceral afferents (Christianson *et al*. 2006; Christianson *et al*. 2007). We found excellent labelling of dorsal root ganglion and pelvic ganglion neurons up to 14 days post‐injection, but successfully visualised sensory axons within the pelvic nerve only at the shortest transport time (4 days). This is consistent with CTB uptake from the injection site occurring over only the first few days, and the CTB subsequently being transported by but not stored within the axon.

By visualising CTB‐labelled axons in the pelvic nerve, we did not detect a difference in location of sensory axons innervating each of the three LUT regions (bladder body, bladder trigone, urethra), but found that sensory axons innervating the LUT were completely segregated from those innervating the large intestine (rectum). This remarkable anatomical distinction may enable selective manipulation of one or other functional pathway within this complex system. It would be of great interest to learn whether the preganglionic and sympathetic components of the pelvic nerve show similar spatial segregation based on their organ function. Many studies have demonstrated a small group of sensory neurons that innervate both the bladder and the colon (Keast and de Groat, 1992; Christianson *et al*. 2007; Pan *et al*. 2010; Ustinova *et al*. 2010; Yoshikawa *et al*. 2015; Grundy and Brierley, 2018). These dichotomising axons may be located across all the fascicles, e.g. they may comprise the very small population of CTB axons that were found in fascicles 1 and 2 after LUT injection or fascicles 3–5 after rectum injection. We also found that many but not all CTB sensory axons were CGRP‐positive, irrespective of their organ projection. These observations are supported by ultrastructural studies of the pelvic nerve following dorsal root ganglionectomy, which determined that approximately one‐third of all pelvic nerve axons are sensory, most of which are unmyelinated (Hulsebosch and Coggeshall, 1982).

We found that each fascicle contained each of the three major axon classes present in the pelvic nerve (sensory, parasympathetic preganglionic and sympathetic postganglionic). Specific patterns of aggregation were more difficult to discern but could be identified for CGRP‐positive sensory axons, that were more strongly aggregated to one region within each of the smaller fascicles. Sympathetic noradrenergic axons were clearly distinguished, not only by their immunoreactivity to TH but also their smooth staining pattern. These axons were present in all fascicles but more prevalent in fascicles innervating the rectum. We could not detect TH expression in any CTB axons, confirming that the selective fascicular distribution of CTB related to sensory function. Visualisation of preganglionic axons was generally less successful, so we could not determine by subtraction how many non‐peptidergic sensory axons were in the pelvic nerve, as CGRP‐, TH‐ and ChAT‐negative axons could also be poorly labelled preganglionic axons.

**Fig. 7:**
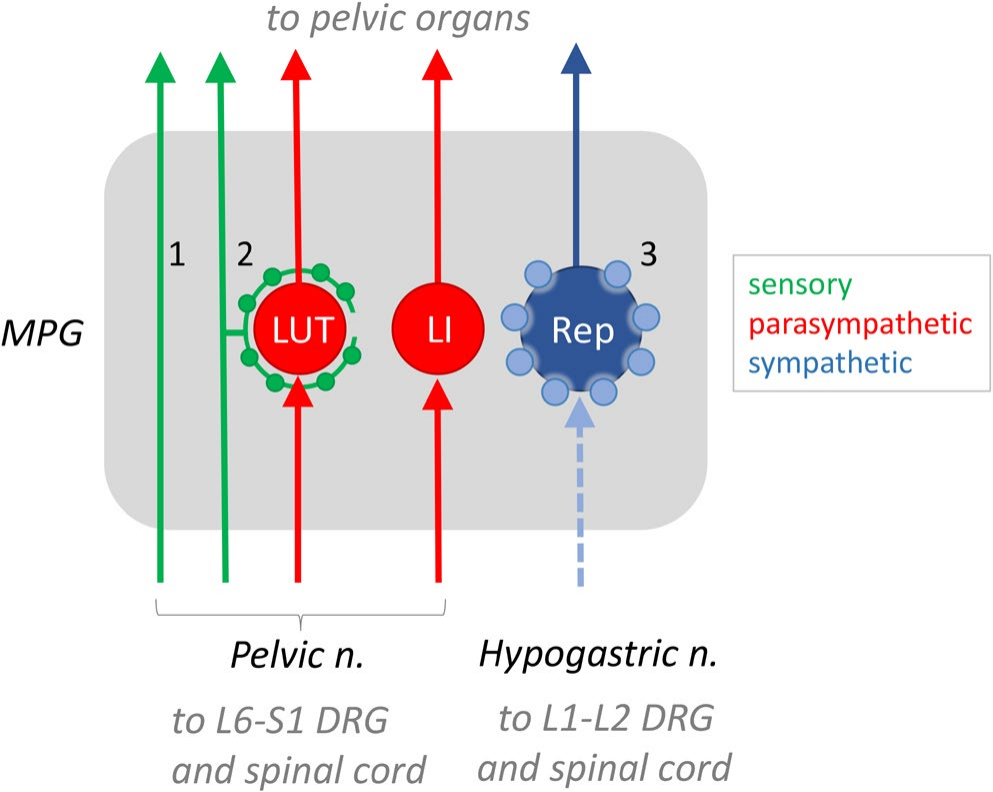
Schematic illustrating the three primary structures expressing calcitonin gene‐related peptide (CGRP) in the male rat major pelvic ganglion (MPG), deduced from retrograde tracing and immunohistochemical analyses. The schematic also shows preganglionic axons that project in the pelvic and hypogastric nerves and innervate MPG neurons. (1) Many CGRP‐positive sensory axons travel across the MPG, exiting to project to the pelvic organs. (2) Some CGRP‐positive sensory axons have collaterals that surround cholinergic MPG neurons, prior to projecting to pelvic organs. Cholinergic neurons projecting to the lower urinary tract (LUT), but not the large intestine (LI), are supplied by these collaterals. Many of the axons of types 1 and 2 are substance P (SP)‐positive, but they do not express vesicular acetylcholine transporter (VAChT). (3) CGRP‐positive varicosities surround many noradrenergic neurons that innervate the reproductive organs (Rep); these cell bodies are larger than the cholinergic neurons. These varicosities express VAChT but not SP and are deduced to originate from lumbar sympathetic preganglionic axons projecting in the hypogastric nerve, shown here as a broken line to indicate that CGRP immunoreactivity is not visible in these preganglionic axons, only in their terminal varicosities. We have shown the origin of the peptidergic sensory nerves as the pelvic nerve [i.e. L6‐S1 dorsal root ganglia (DRG)] but a minority will originate from L1‐L2 DRG, projecting to the MPG via the hypogastric nerve. [Colour figure can be viewed at wileyonlinelibrary.com]

The present study extends our understanding of sacral visceral sensory neurons by examining the projections of CGRP axons from their point of merging with the MPG. The results of these analyses are summarised in Fig. 7. Our study was conducted in whole‐thickness ganglion preparations, enabling visualisation of structures that would be difficult to identify in sections. In particular, continuous tracts of CGRP axons could be followed across the surface of the ganglion to exit in the nerves that carry sensory and motor axons to the pelvic organs. This direct trajectory had not previously been identified, with earlier studies on sectioned MPG focusing on sensory axons entering the mass of ganglion neurons. This raises the question of the mechanisms driving some CGRP axons to penetrate the ganglion tissue to apparently innervate particular neurons, but others to traverse its surface. By co‐labelling for another peptide commonly associated with visceral afferents, SP, we determined that intra‐ganglionic CGRP axons were of two types, only one of which was sensory. The sensory type, identified by its lack of VAChT expression and common co‐expression with SP, was associated exclusively with cholinergic neurons. Many of these cholinergic neurons project to the LUT but none was identified as projecting to the rectum. Because tracing studies do not label the total population of neurons projecting to a particular organ, we cannot determine whether all of these sensory associations are associated with LUT neurons or if a small population of rectum‐projecting neurons are also supplied by sensory axons. In this study, we did not specifically investigate MPG neurons innervating reproductive organs.

Close associations between sensory axons and autonomic ganglion neurons have been reported in several sympathetic ganglia (Hökfelt *et al*. 1977; Kondo and Yui, 1981; Matthews and Cuello, 1982; Matthews and Cuello, 1984; Matthews *et al*. 1987; Stapelfeldt and Szurszewski, 1989). The function of these associations has been investigated in most depth in prevertebral sympathetic ganglia, where SP release is implicated in modulation of ganglionic transmission (Dun and Jiang, 1982; Stapelfeldt and Szurszewski, 1989), although a similar mechanism has also been reported in parasympathetic ganglia (Myers *et al*. 1996). Axons immunoreactive for SP or CGRP, assumed to originate from primary afferent neurons, have also been reported for the pelvic ganglia of rat, cat and guinea pig (Dalsgaard *et al*. 1982; Dail and Dziurzynski, 1985; Papka and McNeill, 1992). In rat MPG these SP axons are present at much lower density than in sympathetic ganglia, estimated as ‘innervating’ only 10%–20% of the total MPG ganglion cell population (Dail and Dziurzynski, 1985). This concurs with our observations. These SP axons degenerate after pelvic nerve transection, consistent with a sacral sensory origin. Very few if any of these SP axons are likely to originate within the MPG as SP‐positive MPG neurons are rare, even after colchicine treatment (Dail and Dziurzynski, 1985). It is intriguing to consider the potential role of sensory axons within the MPG, especially given their specific targeting to cholinergic, LUT‐projecting neurons that mediate contraction of the bladder smooth muscle and relaxation of the muscle of the proximal urethra (Persson *et al*. 1998; de Groat and Yoshimura, 2015). Upregulated peptidergic sensory signalling has been implicated in several types of bladder pathophysiology (e.g. inflammation, spinal cord injury) (Vizzard, 2001; Dickson *et al*. 2006; Zinck *et al*. 2007), raising the possibility of concurrent effects on cholinergic ganglion cell function. These sensory associations with LUT‐projecting MPG neurons also have the potential to be influenced by neuromodulation devices that target visceral sensory pathways.

A second type of CGRP axon identified in the MPG is unlikely to be sensory, based on VAChT expression and structural similarity to terminals of spinal preganglionic neurons in the MPG (Keast, 1995; Eastham *et al*. 2015). These CGRP axons were deduced to be a specific subtype of sympathetic preganglionic axons, as they were associated exclusively with noradrenergic neurons. They were not associated with LUT‐ or rectum‐projecting MPG neurons so are deduced to primarily innervate neurons regulating reproductive organs. In the periphery, CGRP immunoreactivity is often considered synonymous with sensory nerves but our observations support a broader expression profile within the pelvic pathways. Other groups have identified CGRP expression in the terminals of subgroups of autonomic preganglionic neurons (Lee *et al*. 1987; Yamamoto *et al*. 1989; Grkovic *et al*. 1999); the function of CGRP at these terminals is unknown. CGRP immunoreactivity has not been commonly identified in the somata of preganglionic neurons without an experimental manipulation such as colchicine treatment (Yamamoto *et al*. 1989), but its expression has been deduced on the basis of selective denervation and immunohistochemical approaches (e.g. VAChT colocalisation; Eastham *et al*. 2015). An earlier report that many lumbar and sacral preganglionic neurons projecting to the rat MPG express CGRP (Senba and Tohyama, 1988) identified two distinct structures of CGRP axons in the MPG, matching those in the present study. However, they found CGRP strongly expressed by both sympathetic and parasympathetic preganglionic pathways, contrasting with our observation of more specific localisation to sympathetic neurons. Several peptides have previously been identified within preganglionic terminals in the MPG, but most are strongly associated with parasympathetic (sacral) rather than sympathetic (lumbar) pathways (Keast, 1994). Identification of specific subclasses of lumbar preganglionic neurons that target functionally distinct neurons may provide a valuable tool to specifically manipulate a particular subset of neurons rather than all pelvic sympathetic pathways at once.

The outcomes of the present study in rat pelvic nerve raise the possibility of functional separation between components of the human pelvic splanchnic nerves. These nerves are composed of several groups of fibres, mainly emerging from the anterior rami of the third and fourth sacral nerve; in a minority of cases, a small contribution arises from S2 and S5 (Donker, 1986). The pelvic splanchnic nerves commonly have five or six branches, each of a distinct size (Schlyvitsch and Kosintzev, 1939; Donker, 1986), although a more recent study described a greater number of branches (Jang *et al*. 2015). Intra‐operative stimulation studies support the concept of functional segregation within these elements (Possover *et al*. 2005; Possover *et al*. 2007). Specifically, this study focused on two groups of fibres, emerging proximally and distally from the anterior sacral roots of S2 and S3, and showed that it was possible to quite selectively drive an increase in rectal or bladder pressure, depending on whether the proximal (rectal) or distal (bladder) fibres were stimulated. This outcome shows an intriguing parallel with the functional segregation we identified in the rat pelvic nerve. Increasing knowledge of the structural components of each group of fibres and their branches would greatly inform neuromodulation strategies to increase efficacy and reduce off‐target effects.

Limited immunohistochemical studies have been performed on the composition of human pelvic splanchnic nerves. The presence of noradrenergic axons has been demonstrated in adult (Jang *et al*. 2015) and fetal (Alsaid *et al*. 2009) pelvic splanchnic nerve. Preganglionic axons have potentially been identified by immunoreactivity for neuronal nitric oxide synthase (Jang *et al*. 2015) or VAChT (Alsaid *et al*. 2009), which reveal fewer axons than the TH population. These methods may underestimate the preganglionic axons as these proteins are usually present at higher levels in axon terminals rather than axon tracts, and nitric oxide synthase may not be expressed by all preganglionic neurons. To our knowledge, sensory markers have not been examined in the adult or fetal pelvic splanchnic nerves, although they have been reported in the bladder and rectum projection from the fetal inferior hypogastric plexus (Bertrand *et al*. 2016). All of these observations in human tissues (adults or foetuses) should be interpreted with caution because of small sample size and the high level of anatomical variation in the primary structures of this part of the nervous system (Schlyvitsch and Kosintzev, 1939).

In conclusion, the present study provides new insights into the structure of a major peripheral nerve tract, the pelvic nerve, which carries the majority of motor and sensory innervation to the urogenital organs and a substantial component of the extrinsic innervation of the lower bowel. We have also identified sensory associations with pelvic autonomic neurons that are targeted to LUT pathways. Together, these studies reveal that functionally distinct projections within the pelvic nerve have unique structural properties. These properties will be valuable for modelling and modulating the sacral nervous system.

## CONFLICT OF INTEREST

None declared.

## AUTHOR CONTRIBUTIONS

M. Bertrand: contributions to concept/design; acquisition of data; data analysis/interpretation; drafting of the manuscript; critical revision of the manuscript; approval of the article. N. Korajkic: contributions to concept/design; acquisition of data; data analysis/interpretation; critical revision of the manuscript; approval of the article. P. Osborne: contributions to concept/design; data analysis/interpretation; critical revision of the manuscript; approval of the article. J. Keast: contributions to concept/design; acquisition of data; data analysis/interpretation; drafting of the manuscript; critical revision of the manuscript; approval of the article.
